# Exact *p*-values for global network alignments via combinatorial analysis of shared GO terms: *REFANGO*: *R*igorous *E*valuation of *F*unctional *A*lignments of *N*etworks using *G*ene *O*ntology

**DOI:** 10.1007/s00285-024-02058-z

**Published:** 2024-03-29

**Authors:** Wayne B. Hayes

**Affiliations:** Department of Computer Science, UC Irvine, Irvine, USA

**Keywords:** Network alignment, Gene Ontology, GO terms, 05-04, 05C30, 68R05, 68R10, 92-08, 92C15, 92C37, 92C40, 92C42

## Abstract

Network alignment aims to uncover topologically similar regions in the protein–protein interaction (PPI) networks of two or more species under the assumption that topologically similar regions tend to perform similar functions. Although there exist a plethora of both network alignment algorithms and measures of topological similarity, currently no “gold standard” exists for evaluating how well either is able to uncover functionally similar regions. Here we propose a formal, mathematically and statistically rigorous method for evaluating the statistical significance of shared GO terms in a global, 1-to-1 alignment between two PPI networks. Given an alignment in which *k* aligned protein pairs share a particular GO term *g*, we use a combinatorial argument to precisely quantify the *p*-value of that alignment with respect to *g* compared to a random alignment. The *p*-value of the alignment with respect to *all* GO terms, including their inter-relationships, is approximated using the *Empirical Brown’s Method*. We note that, just as with BLAST’s *p*-values, this method is *not* designed to guide an alignment algorithm towards a solution; instead, just as with BLAST, an alignment is guided by a *scoring matrix or function*; the *p*-values herein are computed *after the fact*, providing independent feedback to the user on the *biological* quality of the alignment that was generated by optimizing the scoring function. Importantly, we demonstrate that among all GO-based measures of network alignments, ours is the only one that correlates with the precision of GO annotation *predictions*, paving the way for network alignment-based protein function prediction.

## Introduction and motivation

### Background

Network alignment aims to uncover similar network connection patterns between two or more networks under the assumption that common network topology (which may be easily observable) correlates with common function (which is more difficult to observe). Network alignment algorithms abound and their number is increasing rapidly; see for example Table 1 and recent surveys (Kuchaiev et al. 2010; Mamano and Hayes 2017; Clark and Kalita 2014, 2015; Crawford et al. 2015; Faisal et al. 2015; Guzzi and Milenković 2017; Balomenos et al. 2015). While most practitioners agree on the *goal* of network alignment, in order to test various algorithms against each other for the ability to recover functional similarity, one needs a way to evaluate the functional similarity uncovered by a given network alignment. Unfortunately, there are almost as many ways to evaluate an alignment as there are alignment algorithms.

One of the most common methods for evaluating the biological significance of an alignment involves using the Gene Ontology’s (GO) term hierarchy (The Gene Ontology Consortium 2008). There are several mathematical/statistical complications that arise when attempting to evaluate an alignment using GO terms:

- Most GO terms have inter-dependencies with many other GO terms via the GO hierarchy (Pesquita et al. 2009).
- Most genes and proteins have more than one GO annotation, and it is difficult to create a measure that correctly evaluates similarity between two proteins with different sets of GO terms that only partially overlap.
- Since most GO terms annotate many proteins, it is nontrivial to compute the significance of aligning a set of protein pairs while accounting for both the frequency and inter-relationships between GO terms that may appear in multiple pairs across the set of aligned pairs.
- Even given just one GO term *g*, it is nontrivial to compute the statistical significance of the event that *k* protein pairs in the alignment share *g*.

In this paper we deal *only* with the last issue: given a particular global alignment between a pair of networks in which *k* aligned protein pairs share a specific GO term *g*, we compute the exact *p*-value that a random alignment would have *k* such aligned pairs. The good news is that, once an exact *p*-value is known for each GO term *g*, the *Empirical Brown’s Method* (Poole et al. 2016) can be used to approximately account for the other complications above, which from a statistical significance standpoint simply manifest as correlations between the annotations of different GO terms..

Additionally, there are non-mathematical considerations when using GO terms: protein function is ultimately determined experimentally, so there is always experimental uncertainty involved in claiming that a certain protein should be annotated with a particular GO term; molecular and cellular biology is far from being fully understood, and so the GO term hierarchy itself is in constant flux, with new GO terms introduced as completely novel functions are discovered, or GO terms being merged or split or even deleted as the functional hierarchy is re-evaluated; and different authors may disagree on which GO terms are important, reliable, etc. While these are obviously important scientific considerations, they are beyond the scope of this paper and we will not discuss them further.

### Brief survey of existing GO-based measures of network alignments

For consistency, we will use the following definitions for all methods described below. Let *C* be a “cluster” of aligned proteins—this is a set of proteins that the network alignment, however constructed, has deemed to be “similar” to each other; the cardinality of the set is $|C|\ge 0$, and it can contain proteins from the same network, or different networks. Some methods (such as ours described later) must have $|C|=2$—in which case we called it an “aligned pair” of proteins—but not all methods demand this. Let the PPI networks be $G_i=(V_i,E_i)$ where $i\ge 2$, $V_i$ is the set of nodes (proteins) in network $G_i$, and $E_i$ is its edge list—the set of interactions. Individual nodes (proteins) in a network may be referred to as *u*, *v* (possibly with subscripts), etc. An individual GO term is referred to as *g*, and $\lambda ^g$ is the number of proteins that *g* annotates in a given network. Given proteins *u* and *v*, let $A_u$ and $A_v$ be the set of GO terms that annotate them, respectively; thus *u* is annotated with $|A_u|$ distinct GO terms, and *v* with $|A_v|$.

To motivate our work, we first describe, to our knowledge, an exhaustive list of GO-based methods used to evaluate functional similarity in network alignments (cf. Table 1), and then describe some of their major drawbacks.

**Table 1:** Sample of published network alignment algorithm names, with their citation, year, and the method(s) they used to evaluate functional similarity

Algo	Eval							
	Year	Jac	Com	MNE	Res	Sch	Enr	m-sim
Graemlin (Flannick et al. 2006)	2006	.	.	.	.	.	${\checkmark }$	${\checkmark }$
IsoRank (Singh et al. 2008)	2008	${\checkmark }$	.	.	.	.	${\checkmark }$	.
GRAAL (Kuchaiev et al. 2010)	2010	.	${\checkmark }$	.	.	.	.	.
H-GRAAL (Milenković et al. 2010)	2010	.	${\checkmark }$	.	.	.	.	.
MIGRAAL (Kuchaiev and Pržulj 2011)	2011	.	${\checkmark }$	.	.	.	.	.
GHOST (Patro and Kingsford 2012)	2012	.	.	.	${\checkmark }$	.	.	.
NETAL (Neyshabur et al. 2013)	2013	.	${\checkmark }$	.	.	${\checkmark }$	.	.
SPINAL (Aladağ and Erten 2013)	2013	${\checkmark }$	.	.	.	.	.	.
PIswap (Chindelevitch et al. 2013)	2013	${\checkmark }$	.	.	.	.	.	.
BEAMS (Alkan and Erten 2014)	2014	${\checkmark }$	.	.	.	.	.	.
NetCoffee (Hu et al. 2014)	2014	.	.	.	.	${\checkmark }$	.	.
MAGNA (Saraph and Milenković 2014)	2014	.	${\checkmark }$	${\checkmark }$	.	.	.	.
HubAlign (Hashemifar and Xu 2014)	2014	${\checkmark }$	.	.	${\checkmark }$	${\checkmark }$	.	.
SiPAN (Alkan and Erten 2015)	2015	${\checkmark }$	.	.	.	.	.	.
FUSE (Gligorijević et al. 2015)	2015	${\checkmark }$	.	${\checkmark }$	.	.	.	.
MeAlign (Gong et al. 2015)	2015	${\checkmark }$	.	.	.	.	.	.
OptNetAlign (Clark and Kalita 2015)	2015	${\checkmark }$	${\checkmark }$	.	.	.	.	.
LGRAAL (Malod-Dognin and Pržulj 2015)	2015	.	.	.	${\checkmark }$	.	.	.
WAVE (Sun et al. 2015)	2015	.	.	${\checkmark }$	.	.	${\checkmark }$	.
HGA (Xie et al. 2016)	2016	.	${\checkmark }$	${\checkmark }$	.	.	.	.
DirectedGr (Sarajlić et al. 2016)	2016	.	.	.	.	.	${\checkmark }$	.
ModuleAlign (Hashemifar et al. 2016)	2016	.	.	.	.	${\checkmark }$	.	.
ConvexAlign (Hashemifar et al. 2016)	2016	${\checkmark }$	.	${\checkmark }$	.	${\checkmark }$	.	.
PROPER (Kazemi et al. 2016)	2016	${\checkmark }$	.	.	.	.	.	.
GMalign (Zhu et al. 2017)	2017	${\checkmark }$	.	.	${\checkmark }$	.	.	.
INDEX (Mir et al. 2017)	2017	${\checkmark }$	${\checkmark }$	.	${\checkmark }$	.	.	.
Ulign (Malod-Dognin et al. 2017)	2017	.	.	.	.	.	${\checkmark }$	.
SANA (Mamano and Hayes 2017)	2017	.	.	.	${\checkmark }$	.	.	.
GLalign (Milano et al. 2018)	2018	.	.	.	${\checkmark }$	.	.	.
PrimAlign (Kalecky and Cho 2018)	2018	${\checkmark }$	.	.	.	.	.	.
IBNAL (Elmsallati et al. 2018)	2018	${\checkmark }$	.	.	.	.	.	.
MAPPIN (Djeddi et al. 2018)	2018	${\checkmark }$	.	${\checkmark }$	.	${\checkmark }$	.	.
multiMagna (Vijayan and Milenković 2018)	2018	.	${\checkmark }$	${\checkmark }$	.	.	.	.
MUNK (Fan et al. 2019)	2019	${\checkmark }$	.	.	.	.	.	${\checkmark }$

The rows are sorted by publication year; the columns are sorted by popularity of evaluation measure. Header Legend: Jac = Jaccard Similarity (called “GOC” and “FC” by some authors); Com = number of “common” GO terms in the cluster; MNE = Mean Normalized Entropy; Res = Resnik (Resnik 1995, 1999); Sch = Schlicker’s method (Schlicker et al. 2006); Enr = Enrichment of GO terms in a cluster compared to average cluster; *m*-sim = similarity using only GO terms with frequency ($\lambda$ in our notation) less than *m*

*Jaccard Similarity (aka Functional or GO consistency)* The Jaccard similarity is the most popular method according to Table 1, though it has variously been called *GO Correctness* or *Consistency* (GOC), as well as *Functional Correctness/Consistency* (FC). Formally, given node $u\in V_1$ aligned to $v\in V_2$, let $A_u,A_v$ be the set of GO terms annotating *u*, *v*, respectively. Then the Jaccard/GOC/FC between *u* and *v* is defined as

1 $$\begin{aligned} \text{ FC }(u,v)\equiv \text{ GOC }(u,v)\equiv \text{ Jaccard }(u,v)\equiv \frac{|A_u \cap A_v|}{|A_u \cup A_v|}. \end{aligned}$$

Given this similarity across all aligned pairs of proteins, the FC score of the entire alignment is the mean FC across all aligned pairs.

*Common GO terms* Given a network alignment, choose an integer threshold *h* (typically 1–5), and count the number of aligned pairs that have at least *h* GO terms in common. No effort is made to account for the annotation frequencies ($\lambda$ values in our terminology), or location in the hierarchy, of any GO term.

*Entropy* Given a cluster of proteins *C* in which *d* GO terms $\{g_1,\ldots ,g_d\}$ appear at least once across all the proteins in *C*, the entropy is defined as $H(C)=-\sum _{i=1}^d p_i\log p_i,$ where $p_i$ is the fraction of all proteins in *C* that are annotated with GO term $g_i$. Entropy is always non-negative and lower values are better. The *normalized entropy* is $N(C)=H(C)/d$. Alignments can then be scored using *Mean Entropy* (ME) or *Mean Normalized Entropy* (MNE), which is just the appropriate mean across all clusters *C*. The first network alignment algorithm to use MNE was IsoRankN (Liao et al. 2009).

*Resnik* Resnik’s measure of semantic similarity (Resnik 1995, 1999) was originally designed only to evaluate the similarity between two terms in an ontology by finding their *most informative common ancestor* in the hierarchy, and using an information-theoretic argument to compute their common information. Later it was extended to measure similarity between gene products (such as proteins) with multiple GO annotations, by taking some sort of mean or maximum between the GO terms of two proteins (see, e.g., Schlicker et al. 2006; Pesquita et al. 2008, 2009).

*Schlicker’s method* is a variant of Resnik’s measure tailored specifically to genes and gene products (Schlicker et al. 2006).

*Enrichment* has been defined in various ways but usually measures whether the shared annotations to genes or proteins in a “set of interest” is “enriched” beyond what is expected compared to a “background” rate of annotations. While enrichment is one of the few methods that accounts for the total annotation frequency across the network, enrichment analysis would still need to be done cluster-by-cluster, and so would suffer the same problem as all other cluster-by-cluster methods.

*m*-*sim* This measure is used only by Graemlin (Flannick et al. 2006) and MUNK (Fan et al. 2019); the latter technically is not a network alignment algorithm, though it is designed to find functionally similar genes or proteins between species. This is the only method from Table 1 that takes into account the annotation frequency $\lambda ^g$ of a GO term *g* across the entire network, by using only GO terms with frequency below some threshold *m*.

### Problems with existing methods

Table 1 presents a list of alignment papers and the measures they use to evaluate functional similarity. Without exception, all of these methods evaluate each pair of aligned nodes individually, and then take the average across pairs. (Some methods are not 1-to-1 and so the “pair” of aligned nodes we discuss must be generalized to a *cluster* of aligned nodes, but this generalization does not negate our point.)

We are aware of no existing methods that consider the alignment from the perspective of one GO term’s performance globally across all clusters, rather than looking cluster-by-cluster. The result is that all of these methods suffer major drawbacks.

#### Cluster-by-cluster analyses fail to account for completely unmatched GO terms

There is a crucially important case that is implicitly ignored by methods that evaluate GO-based significance of network alignments by simply taking the mean of a score evaluated cluster-by-cluster. This case is alluded to by phrases such as “consider the GO terms shared by a pair of aligned proteins...”. The problem is when there is a GO term *g* that exists in both networks, but no pair of aligned proteins share it. Then the “consider...” phrase above implicitly misses the fact that *g* *could* have been shared by some aligned protein pairs, but was not.^1^ Unless taken care of explicitly, the alignment evaluation fails to penalize the alignment for failing to provide any matches for GO term *g*. In contrast, our method (below) is correctly penalized for such cases: any GO term *g* that occurs in both networks but is not matched by any aligned pair of nodes receives the appropriate penalty of a *p*-value with little statistical significance. Unfortunately, since many existing publications ignore this case, many published *p*-values claim far more statistical significance than actually exists.

#### Average cluster scores do not distinguish good alignments from random ones

The biggest problem with evaluating a network alignment by taking the mean across cluster-based measures is that such measures do not scale even remotely monotonically with statistical significance. To give an explicit example, we’ll use the Jaccard Similarity, which is the most popular based on Table 1.

Consider the following simple system: network *G* has $n=1000$ nodes. Each node is annotated with exactly one GO term. The first 100 nodes $v_1,v_2,\ldots ,v_{100}$ are each individually annotated with their own unique GO term, with names $\{g_1,g_2,\ldots ,g_{99},g_{100}\}$, respectively. We will refer to these as the “specific” nodes, in the sense that their functions are all relatively unique and well-specified since they all have different GO terms. The remaining 900 nodes are all annotated with the same GO term—say $g_0$. We will refer to these as the “common” nodes, and their functions are less well-specified, and likely less well-understood, since all we can say is that they all participate in some high-level, likely vague and not well specified function. From the network alignment perspective, correctly aligning specific nodes is far more informative than aligning common nodes to each other, since identifying individual proteins with well-specified functions is usually more desirable than aligning one common node to another one.

For simplicity, we will align *G* to itself, and assume that all 101 of the GO terms are *independent*, so that the *p*-value of the entire alignment is the product of the *p*-values across the 101 GO terms.^2^ Then, every pair of aligned nodes constitutes a *cluster*, and the only possible per-cluster FC scores are 0 and 1, so that the mean alignment-wide FC score is simply the fraction of node pairs that have FC $=1$.

If an aligned pair of nodes are annotated with the same GO term, we call it a “match”. In a random alignment of *G* to itself, each common node has a 90% chance of being aligned with another common node, so that the expected number of matched common nodes is $900\times 0.9 = 810$. On the other hand, each specific node has only a 0.1% chance of being aligned with its one and only match, so that in a random alignment we expect *none* (or very few) of the specific nodes to match. For this example, assume we match 5 more common nodes than expected at random (815 of them), but match none of the specific nodes (as expected). Using the Hypergeometric distribution, the probability of matching 815 or more common nodes (and no specific ones) has probability 0.062—not statistically significant. Thus, the alignment has FC score of 0.815 (815 out of 1000 nodes having FC = 1)—making it look very good—but with a *p*-value of just 0.06.

Now consider a second alignment with the same FC score: here we also match 5 more than the expected number of nodes, but this time they are all *specific* nodes—noting that the expected number is zero; we assume that the common nodes get the 810 expected matches. Thus, the mean FC score across clusters is $(810+5)/1000=0.815$, exactly as in the previous case. By the Hypergeometric distribution, matching 810 or more out of 900 common nodes has a *p*-value of 0.555. However, *each* specific node has probability only $10^{-3}$ of aligning to itself in a random alignment, so the *p*-value of matching 5 of them is $10^{-15}$; the *p*-value of the other 95 not matching is 0.999 each, or 0.91 total. So the total *p*-value of the second alignment is slightly less than $10^{-15}$.

Thus, both alignments have a mean FC of 0.815, yet—to the nearest order-of-magnitude—the first has virtually nil statistical significance, while the second has a *p*-value below $10^{-15}$. From a statistical significance standpoint, the second one is—quite literally—an *astronomically* better alignment. It’s also easy to see that the *p*-value of any alignment that aligns *k* of the “specific” nodes will have a *p*-value of about $10^{-3k}$, which is better than the first case for any $k>0$.

The takeaway message is that any method that evaluates functional significance cluster-by-cluster and then takes the mean across clusters—as do all existing methods—can lead to very misleading conclusions by making near-random alignments look just as good as excellent ones.

#### The problem with ignoring GO terms close to the root of the hierarchy

A common practice (Pesquita et al. 2009) involves arbitrarily ignoring GO terms in the top few levels of the GO hierarchy on the assumption that, when a GO term annotates so many proteins, a protein pair that matches it has little value. A known problem (Pesquita et al. 2009) with this suggestion is the definition of “top few levels”: even GO terms at the same level but different regions of the GO hierarchy can have vastly different values of $\lambda$, so that it is difficult to choose which GO terms to ignore. While there are sometimes valid reasons for ignoring such common GO terms—such as the fact that they may be “catch-all” terms with little meaning or with very low confidence—there may be cases where ignoring them is unjustified.

From the network alignment perspective, ignoring these common GO terms has the opposite problem to that of Sect. 1.3.1 in that, rather than failing to *penalize* a bad alignment, this procedure fails to adequately *reward* alignments that are “good” in the following sense. Assume a GO term *g* annotates 10% of proteins in each network, and that these annotations are not simply low-confidence, “catch-all” GO terms. This can be a substantial number of proteins (e.g., over 1700 in human and almost 700 in mouse), and such a GO term is likely to be high in the hierarchy. However, if a network alignment matches a substantially larger fraction of this plethora of pairs than is expected at random, it is a sign that *large regions* of functional similarity are being correctly aligned to each other, even if individual proteins are not. In other words, perhaps similar pathways are being correctly mapped to each other even if the individual proteins in the pathway are incorrectly mapped. A network alignment that successfully matches such large regions should be rewarded for doing so, but if “common” GO terms are disregarded, this won’t happen.

## Method: GO-term *p*-values by exhaustive enumeration of alignments

### Network alignment and functional similarity

Given two networks $G_1,G_2$, let the node sets $V_1,V_2$ represent $n_1$ and $n_2$ proteins respectively, and the edge sets $E_1,E_2$ represent protein–protein interactions (PPIs). Assuming (without loss of generality) that $n_1\le n_2$, a pairwise global network alignment (PGNA) is a 1-to-1 mapping $f:V_1\rightarrow V_2$ in which every node in $V_1$ is mapped to exactly one node in $V_2$.

Once an alignment is specified, we usually wish to use it to measure, infer, or predict functional similarity between proteins and/or pathways aligned between the two networks. As discussed above, most existing methods perform a cluster-by-cluster analysis and then take a mean across clusters. In addition to the shortcoming already mentioned, taking an average across clusters—aligned node pairs in our case—assumes that each pair is independent of all the others. This is not true because the pairings themselves are inter-dependent via the alignment itself, which is built globally. For example, in a 1-to-1 alignment, each node from each network can appear at most once across the entire alignment, a property which destroys the independence assumption.

Our solution to this problem is to look at an alignment from the viewpoint of one *GO term* at a time, rather than one *aligned pair of proteins* at a time. To that effect, we now describe how to compute the exact *p*-value that exactly *k* aligned protein pairs share a particular GO term *g*.

### Computing the total number of possible alignments

In the following exposition, we must discuss in great detail the combinatoric structure of a given alignment. To aid visualization, we use what I call the “Pegs and Holes” analogy: given networks $G_1, G_2$ with $n_1, n_2$ nodes, we imagine $G_2$’s nodes as $n_2$ identical “holes” drilled into a large board, and $G_1$’s nodes as $n_1$ identical “pegs” that can each fit into any hole. To enforce the global 1-to-1 property, there are two cases:

1. $n_1\le n_2$, so every peg is placed into some hole, leaving $n_2-n_1$ empty holes. There are ${n_2\atopwithdelims ()n_1}$ ways to choose which holes to use, and $n_1!$ ways to place the pegs.
2. $n_1 > n_2$, so every hole is filled with some peg, leaving $n_1-n_2$ pegs unplaced. There are ${n_1 \atopwithdelims ()n_2}$ ways to choose which pegs to place, and $n_2!$ ways to place them.

The above two cases are symmetric and so, without loss of generality, we assume $n_1\le n_2$. Then, the total number of all possible alignments is

2 $$\begin{aligned} {n_2\atopwithdelims ()n_1}n_1! = \frac{n_2!}{(n_2-n_1)!}\equiv P(n_2,n_1). \end{aligned}$$

The function $P(\cdot ,\cdot )$ of Eq. (2) is more commonly known as *k-permutations-of-n*, or *P*(*n*, *k*). However, *P*(*n*, *k*) is usually defined to be zero if $n<k$, whereas we will often need to compute the number of alignments when we don’t know which of the two values is larger. Thus, in this paper, we will adopt a modified permutation function $\pi (n_1,n_2)$ as follows

3 $$\begin{aligned} \pi (n_1,n_2) = \left\{ \begin{array}{cc} P(n_1,n_2), &{} \text{ if } n_1\ge n_2, \\ P(n_2,n_1), &{} \text{ if } n_2> n_1. \end{array} \right. \end{aligned}$$

### Counting alignments with exactly *k* matches

Given a particular GO term *g*, assume *g* annotates $\lambda _1$ pegs and $\lambda _2$ holes. A peg and the hole it sits in are, more technically, a pair of aligned nodes. We say that such a pair “match” with respect to GO term *g* if they are both annotated with *g*. Let ${\underline{\lambda }}=\min (\lambda _1,\lambda _2)$, and ${\overline{\lambda }}=\max (\lambda _1,\lambda _2)$. Given a random 1-to-1 alignment, we are going to compute the probability *p* that exactly *k* pairs of aligned nodes share *g*. In our analogy, this means that exactly *k* pegs—no more, no less—that are annotated with *g* sit in holes that are also annotated with *g*. To do this, we will use a combinatorial argument to enumerate all possible PGNAs that can exist that have exactly *k* matches. Given that number, we simply divide by Eq. (2) to get the probability that a randomly chosen alignment has exactly *k* matches.

#### Special cases

The following are special cases:

1. if $$k>{\underline{\lambda }}$$, then $$p=0$$.
2. if ${\underline{\lambda }}=0$, then $p=1$ if $k=0$ and $p=0$ otherwise.
3. if $\lambda _2=n_2$, then $p=1$ if $k=\lambda _1$, and $p=0$ otherwise.
4. if $\lambda _1>n_2-\lambda _2$ and $k<\lambda _1-(n_2-\lambda _2)$, then $p=0$, otherwise $p>0$ is computed below.

The last case arises when $\lambda _1>n_2-\lambda _2$, which means that there are more annotated pegs than non-annotated holes, necessitating that *at least* $\lambda _1-(n_2-\lambda _2)$ annotated pegs must align with annotated holes. (Recall we are computing the probability of *exactly* *k* aligned pairs sharing *g*, so *k* too small in this case gives $p=0$.)

Below we describe the general case in detail. In broad outline, there are three steps: (i) create the required *k* matches by placing *k* annotated pegs into *k* annotated holes; (ii) arrange to place the remaining annotated pegs away from the annotated holes in order to keep *k* constant; (iii) place any remaining pegs (all of which are non-annotated) in any still-empty holes (some of which may be annotated). In each case we either sum, or multiply, as appropriate, the number of ways to perform the described action. In the end we have counted all the possible ways to create an alignment that has exactly *k* matches.

#### Creating exactly *k* matches

Out of the $\lambda _1$ pegs annotated with *g*, pick $k\le {\underline{\lambda }}$ of them; there are ${\lambda _1 \atopwithdelims ()k}$ ways to do this. We will place these *k* pegs into *k* holes that are also annotated with *g*; there are ${\lambda _2\atopwithdelims ()k}$ ways to pick the holes, and *k*! ways to place the *k* pegs into the *k* holes. Thus, the total number of ways to match exactly *k* pairs of nodes that share *g* is

4 $$\begin{aligned} M_k(\lambda _1,\lambda _2)={\lambda _1 \atopwithdelims ()k} {\lambda _2 \atopwithdelims ()k}k!. \end{aligned}$$

From this point onward, in order to keep *k* constant, we are committed to creating no more matches.

#### Enumerating the ways to use the remaining annotated holes

To ensure that no more node pairs are matched, we need to ensure that none of the remaining $(\lambda _1-k)$ annotated pegs are placed into any of the remaining $(\lambda _2-k)$ annotated holes. Thus, each annotated hole must either remain empty, or take an non-annotated peg. There are $n_1-\lambda _1$ available non-annotated pegs, regardless of the value of *k*. Pick $\mu$ of them. Since these $\mu$ pegs are all non-annotated, they can go into any unoccupied annotated hole without changing *k*. However, there are lower and upper bounds on what $\mu$ can be, as follows:

- $\mu$ can be at most ${\overline{\mu }}\equiv \min (n_1-\lambda _1,\lambda _2-k)$, since $n_1-\lambda _1$ is the total number of non-annotated pegs, and $\lambda _2-k$ is the number of available annotated holes in which to place (some of) them.
- note that we have $n_1-k$ pegs (of both types) remaining to place, and exactly $n_2-\lambda _2$ non-annotated holes, into which some (or all) of the pegs can be placed. By the pigeon hole principle, if $(n_1-k)>(n_2-\lambda _2$), then some of the pegs—and they can only be non-annotated pegs—*must* go into annotated holes. Thus, $\mu$—which refers only to non-annotated pegs—must be at least ${\underline{\mu }}\equiv (n_1-k)-(n_2-\lambda _2)$ if $(n_1-k)>(n_2-\lambda _2)$; otherwise ${\underline{\mu }}=0$.

#### Distributing the remaining pegs

For any ${\underline{\mu }}\le \mu \le {\overline{\mu }}$, we need to count how many alignments can be built when $\mu$ non-annotated pegs are placed into the $\lambda _2-k$ available annotated holes, as well as what happens to all the remaining pegs. The process is as follows.

1. There are ${n_1-\lambda _1 \atopwithdelims ()\mu }$ ways to choose $\mu$ non-annotated pegs, and $\pi (\lambda _2-k,\mu )$ ways to align them with the open annotated holes. To simplify notation note that $n_1,n_2,\lambda _1,\lambda _2$ are all fixed; thus, let $\gamma _k(\mu )={n_1-\lambda _1 \atopwithdelims ()\mu }\pi (\lambda _2-k,\mu )$.
2. Recall that there are still $\lambda _1-k$ annotated pegs to be placed, and that they must be placed into non-annotated holes, so we must “reserve” $\lambda _1-k$ non-annotated holes, which will be further accounted for below.
3. Once $\mu$ annotated holes are filled with non-annotated pegs, the rest of the annotated holes must remain empty; this leaves $n_1-\lambda _1-\mu$ non-annotated pegs to go into the $n_2-\lambda _2$ non-annotated holes. Keeping in mind the “reservation” above, there are $n_2-\lambda _2-(\lambda _1-k)$ available non-annotated holes. There are ${n_2-\lambda _2 \atopwithdelims ()\lambda _1-k}$ ways to choose which holes to use while reserving $\lambda _1-k$ of them, and $\pi (n_1-\lambda _1-\mu ,n_2-\lambda _2-(\lambda _1-k))$ ways to place the pegs into the chosen holes; let $\delta _k(\mu )={n_2-\lambda _2 \atopwithdelims ()\lambda _1-k} \pi (n_1-\lambda _1-\mu ,n_2-\lambda _2-(\lambda _1-k))$.
4. Finally, we place the remaining $\lambda _1-k$ annotated pegs into the reserved holes of the same number; there are $(\lambda _1-k)!$ ways to do this.

#### Summing the unmatched region of the alignment

Combining all of the above for fixed $\mu$ and then summing over all possible $\mu$, the total number of ways that $n_1-\lambda _1$ non-annotated pegs can be used to (partially or wholly) fill $\lambda _2-k$ annotated holes, and then use all the remaining pegs and holes in a manner consistent with keeping *k* constant, is

5 $$\begin{aligned} U_k(\lambda _1,\lambda _2) \equiv (\lambda _1-k)! \sum _{\mu ={\underline{\mu }}}^{{\overline{\mu }}} \gamma _k(\mu ) \delta _k(\mu ). \end{aligned}$$

#### Final tally for exactly *k* matches

Combining Eq.s (4) and (5), the total number of alignments in which exactly *k* aligned node pairs share GO term *g* is

6 $$\begin{aligned} C_k(\lambda _1,\lambda _2)\equiv M_k(\lambda _1,\lambda _2) U_k(\lambda _1,\lambda _2). \end{aligned}$$

### The probability of an alignment with exactly *k* matches

Equation (6) counts all possible alignments in which exactly *k* aligned node pairs share GO term *g*. To get the probability $p_k$ of the same event, we divide by Eq. (2):

7 $$\begin{aligned} p^g_k(n_1,n_2,\lambda ^g_1,\lambda ^g_2) = \frac{C^g_k(\lambda ^g_1,\lambda ^g_2)}{\pi (n_1,n_2)}, \end{aligned}$$

where a superscript *g* has been added as appropriate to denote that this probability is specifically tied to GO term *g*.

Note this refers to *exactly* *k* matches. To measure the statistical significance of *m* matches, we sum Eq. (7) for *k* from *m* to ${\underline{\lambda }}^g$.

### Efficiently dealing with huge numbers

Though technically it is only an implementation detail, it is important to briefly discuss how to deal with the astronomically huge numbers involved in these calculations. Typical modern biological networks can have thousands to tens of thousands of nodes, and some GO terms annotate thousands of genes in each network. For example, in BioGRID 3.4.164 that we use below, the two biggest PPI networks in terms of number of nodes are *H. sapiens* and *A. thaliana*, which contain exactly 17,200 and 9,364 unique proteins, respectively, that are involved in physical interactions. Equation (2) in this case is approximately $10^{38270}$—an integer with over 38,000 digits in base-10, which is far above the values typically representable on modern hardware. Luckily, its logarithm is easy to represent in double precision floating point, and so all of the multiplications herein can be computed as the floating-point sum of logarithms. The sole complication is the summation in Eq. (5), which is a sum of *values*, not logarithms. We use the following trick. Given two numbers *a* and *b*, assume we have at our disposal only their logarithms, $\alpha =\log (a)$ and $\beta =\log (b)$. Our goal is to estimate $\log (a+b)$. Without loss of generality, assume $a\le b$. Then,

8 $$\begin{aligned} \log (a+b)= & {} \beta + \log (1+a/b) \end{aligned}$$

9 $$\begin{aligned}= & {} \beta + \log (1+e^{\alpha -\beta }) \end{aligned}$$

10 $$\begin{aligned}= & {} \beta + L(e^{\alpha -\beta }), \end{aligned}$$

where *L*(*x*) is some function that can provide an accurate estimate of $\log (1+x)$ for any $|x|\le 1$. One must be careful because if |*x*| is below the machine epsilon ($\approx 10^{-16}$ in double precision), then $1+x$ evaluates to 1 because *x* is rounded away, and a direct evaluation of the expression $\log (1+x)$ gives zero. The solution is not hard: the built-in library function for log can evaluate $\log (1+x)$ with sufficient accuracy if $|x|>10^{-6}$; for smaller values of |*x*|, we explicitly invoke the Taylor series, which is extremely accurate for small values of |*x*|. We have tested that this method gives values for $\log (a+b)$ that are accurate to almost machine precision for any $|x|\le 1$.

### Run time

Our algorithm has several steps. Reading the OBO file and constructing the internal representation of the GO hierarchy takes time $O(|GO|^2)$, where |*GO*| is the number of GO terms in the hierarchy; expanding the explicitly listed annotations listed in the GO database for each protein technically takes time $O(|n_1+n_2|\times |GO|^2)$ but practically speaking is much faster since most protiens are annotated only by a few GO terms rather than *all* GO terms ($n_1$ and $n_2$ are the number of proteins in the two networks). The only other loop is through $\mu$ in Eq. 5, which is performed only once and is bounded by $O(\max (n_1,n_2))$.

From a practical standpoint, the runtime is only a minute or two, even though the code is entirely in AWK; converting to C/C++ would make the runtime completely negligible on existing PPI or gene networks.

## Results

### Numerical validation

Staring at $C_k(\lambda _1,\lambda _2)$ in Eq. (6) and tracing back through the equations that define its components, it is not immediately obvious that the $C_k(\lambda _1,\lambda _2)$, when summed over all possible values of *k*, must add up to exactly $\pi (n_1,n_2)$ independent of the choice of $\lambda _1,\lambda _2$. Yet if Eq. (6) is correct, then this must be the case since summing $p_k$ in Eq. (7) across all *k* of must give exactly 1.

In the calculation of $p^g_k$ in Eq. (7), the values of *k* and *g* are fixed. For a fixed *g*, valid values of *k* range from zero to ${\underline{\lambda }}^g$. If our calculations are correct, then the sum across *k* of $p^g_k$ should be exactly 1 for any fixed $g,n_1,n_2,\lambda _1,\lambda _2$. We tested this property in the following cases:

1. exhaustively for all $0\le \lambda _1\le n_1$ and $0\le \lambda _2\le n_2$ for all $0\le n_1\le n_2\le 100$;
2. as above but in steps of 10 in $\lambda _i$ and $n_i$ up to $n_2=1,000$;
3. as above but in powers of 2 in $\lambda _i$ and $n_i$ up to $n_2=32,768$;
4. several billion random quadruples of $(n_1,n_2,\lambda _1,\lambda _2)$ with $n_2$ chosen uniformly at random up to 100,000, $n_1$ chosen uniformly at random up to $n_2$, and the $\lambda$’s chosen uniformly at random up to their *n* value.

We found in all cases that the difference from 1 of the sum over *k* of $p^g_k$ was bounded by $10^{-9}$. (Keep in mind that we had access only to the logarithms of the $C_k$; that the actual sum across *k* had to be approximated term-by-term using Eq. (10); that the correct answer in log space is $\log (1)=0$; and that all operations were performing in floating point, which incurs roundoff error.) Furthermore, in any particular case, the numerical (floating-point roundoff) error will be dominated by the sum over $\mu$ in Eq. (5), and so we would expect the error to be smaller (ie., sum closer to 1) when there are fewer terms in Eq. (5). The number of terms is well-approximated by $\min (n_1-\lambda _1,n_2)$. Indeed, we find that if the sum was *S*, then the value $|S-1|/\min (n_1-\lambda _1,n_2)$ has mean $\approx 3\times 10^{-14}$, standard deviation $\approx 3\times 10^{-13}$, and was never observed to exceed $3\times 10^{-12}$.

### Validation against random alignments of real PPI networks

**Table 2:** The 8 largest networks of BioGRID 3.4.164, sorted by node count

Nodes	Common name	Official name	Abbr
17,200	Human	*H. sapiens*	HS
9364	Thale cress	*A. thaliana*	AT
8728	Fruit fly	*D. melanogaster*	DM
6777	Mouse	*M. musculus*	MM
5984	Baker’s yeast	*S. cerevisiae*	SC
3194	Worm	*C. elegans*	CE
2811	Fission yeast	*S. pombe*	SP
2391	Rat	*R. norvegicus*	RN

We downloaded the 8 largest protein–protein interaction networks from release 3.4.164 (August 2018) of BioGRID (cf. Table 2), and the GO database release of the same month. As many authors of network alignment papers do, we then split the GO database into two versions: one with all GO terms, and ones where sequence-based GO terms were disallowed. For each of the ${8 \atopwithdelims ()2}=28$ pairs of networks and for both versions of the GO database, we generated 400 million random alignments, for a total of 22.4 billion random alignments. For each GO term *g*, we observed the integer frequency $\phi ^g_k$ that *g* was shared by exactly *k* proteins when it annotated $\lambda ^g_1$ out of $n_1$ proteins in network $G_1$ and $\lambda ^g_2$ proteins out of $n_2$ in network $G_2$. (Note that formally $\phi _k^g$ has six parameters, $\phi ^g_k(n_1,n_2,\lambda ^g_1,\lambda ^g_2)$, though we often abbreviate it to $\phi ^g_k$ or even just $\phi _k$ or $\phi$ if context is clear.) It is a non-negative integer bounded by the number of random alignments, $N=4\times 10^8$, and dividing it by *N* gives an estimate of the probability that a randomly chosen alignment between $G_1$ and $G_2$ will contain exactly *k* aligned protein pairs that share *g*.

**Fig. 1:**
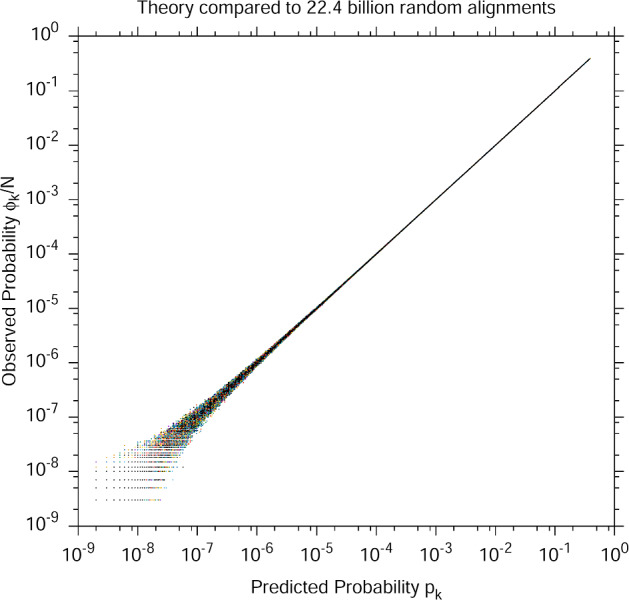
Scatter plot of the observed $\phi _k/N$ versus theoretical $p_k$ probability across 22.4 billion random alignments between pairs of networks from BioGRID 3.4.164. The vertical axis depicts the observed probability of an event, which is the observed frequency $\phi ^g_k(n_1,n_2,\lambda _1,\lambda _2)$ divided by the number of samples $N=4\times 10^8$. The horizontal axis is the value given by Eq. (7) for the parameters of the observation. There are 428,849 observations plotted across all observed values of $n_1,n_2,\lambda ^g_1,\lambda ^g_2,k$

The estimated (ie., observed) probability $\phi ^g_k/N$ can be compared to $p^g_k$ of Eq. (7). Across the 22.4 billion random alignments, we observed 428,849 unique combinations of the six parameters $g,k,n_1,n_2,\lambda ^g_1,\lambda ^g_2$ that formally define $\phi ^g_k$. Figure 1 is a scatter plot of $\phi ^g_k/N$ for all 428,849 of them, versus the theoretical value from Eq. (7). The agreement is excellent. (We note that our Fig. 1 is exactly analogous to Fig. 1 of the paper that introduced BLAST (Altschul et al. 1990), in which the authors compared their statistical model of sequence alignment to computational experiments involving random sequence alignments.)

**Fig. 2:**
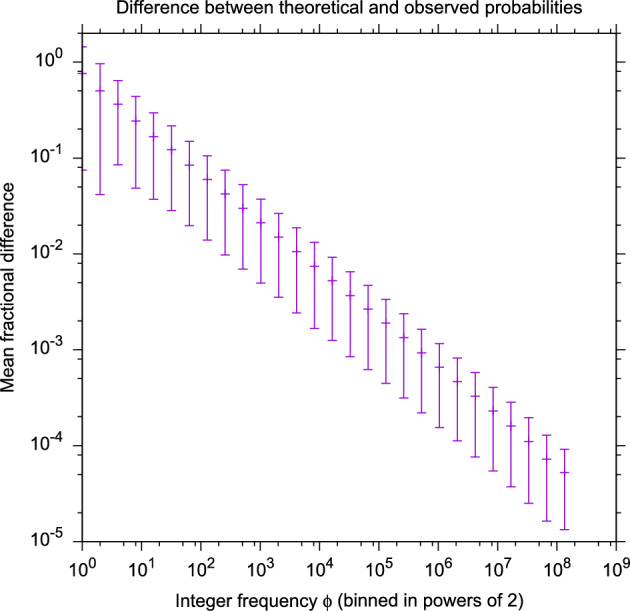
Same data as Fig. 1, except that, for each point, we have computed the distance *D* from 1 of the ratio of observed to predicted probability: $D=|1-\frac{\phi ^g_k/N}{p^g_k}|$. Each observed frequency $\phi ^g_k$ (which we will henceforth abbreviate a $\phi$) is converted to an observed probability $\phi /N$, where *N* is the number of random alignments ($4\times 10^8$) per pair of networks. However, $\phi$ is also the number of samples used to create the observed probability estimate; higher $\phi$ gives a better estimate of the probability. We binned $\phi$ in powers of 2 (ie. the bin is ${\left\lfloor {\log _2(\phi )}\right\rfloor }$, and for each bin plotted the mean and standard deviation of *D*. We see that as the number of samples increases, the ratio approaches 1 as the square root of the number of samples, consistent with sampling noise

The scatter in Fig. 1 increases towards the low end because events with probability near $N^{-1}$ are rarely observed, and so the estimate of their probability contains significant sampling noise. In fact there is “width” to the scatter plot at all values of probability, but it is difficult to observe in Fig. 1. To more clearly see the scatter, we compute the *ratio* of the observed to theoretical values of probability, which will have an expected value of 1 if Eq. (7) is an accurate and unbiased estimator of probability. Figure 2 plots the mean and standard deviation (binned in powers of 2 of the number of samples) of $|1-(\phi ^g_k/N)/p^g_k|$ across all 428,849 observed frequencies, as a function of the number of samples that gave rise to the probability estimate. We can clearly see that the ratio approaches 1 asymptotically with the square root of the number of samples, consistent with sampling noise in $\phi$.

### Demonstration of biological relevance

To demonstrate the biological and scientific relevance of our method, we are going to demonstrate that the quality of a network alignment—as measured by its *p*-value as computed herein—strongly correlates with that alignment’s ability to *predict* new GO term annotations. To do this, we leverage our recent network alignments that were used to predict GO annotations (Wang et al. 2022). In that paper, we used SANA (Mamano and Hayes 2017) to align pairs of BioGRID networks available as of April 2010, and annotated with GO terms also available as of April 2010. In any network alignment where a pair of proteins *u* and *v* were aligned and only one of them (say *u*) was annotated with a particular GO term *g*, we “transferred” the annotation to the other protein (say *v*), resulting in a predicted annotation of *v* with *g*. The predicted annotation was considered *validated* if the annotation of *v* with *g* appeared in the GO database within the following decade—ie., by April 2020.

In the context of this paper, the quality of a pairwise network alignment with respect to a particular GO term *g* is measured using the *p*-value computed in Sect. 2.4. The assumption is that the smaller the *p*-value, the better the alignment. If this is true, then we would expect the precision of predicted annotations to improve as the *p*-value of an alignment gets smaller. However, note that there is a trade-off: as the alignment quality increases by increasing the number of “matched” protein pairs (ie., both proteins are *g*-annotated), the number of *unmatched* *g*-annotated proteins in the source species decreases, decreasing the number of possible predictions that can be made in the target species. Ironically, if every *g*-annotated protein in the source species is already matched with a *g*-annotated protein in the target species, then *no* predictions can be made.

The species used in Wang et al. (2022) included *A. thaliana, C. elegans, D. melanogaster, S. cerevisiae, S. Pombe,* and *H. Sapiens*. We will look at species pairs in which human was the target (ie., a human protein *p* was not annotated with *g* as of April 2010, and it was aligned to a protein *q* from another species that *was* annotated with *g* as of April 2010.) In Wang et al. (2022), we performed 100 network alignments of each pair of species. (This makes sense since SANA is a random search algorithm, and so different runs can produce different alignments, especially if the networks are noisy and/or incomplete—see Wang et al. (2022).) Furthermore, note that in Wang et al. (2022), multiple network alignments of the same pair of species were used to formulate GO term predictions, whereas in this paper we are using the *p*-value of the number of matched GO *g*-annotations in a *single* network alignment to predict new *g*-annotations in the same alignment. Note also that no threshold or any criterion is placed on the pair of proteins being aligned—we *g*-annotate *every* protein in the target species that lacks it if it is aligned to a *g*-annotated protein in the source species.

In total, there were 1185 GO terms represented across all species as of April 2010. However, as alluded to above, if a GO term annotates only a few proteins in the source species (ie., the $\lambda$ value is small—cf. Sect 2.4), then once enough of them are *matched* to get a small *p*-value according to Sect 2.4, there simply won’t be enough unmatched annotations to make a significant number of predictions in the target species. We found that GO terms that produced at least 10 predicted annotations in a single alignment had both adequate predictions to compute a meaningful prediction precision, as well as enough matched GO terms to have a meaningful *p*-value. Thus, a GO term *g* was not considered in an alignment A if that (*g*,A) pair produced fewer that 10 *g*-annotation predictions. After this filtering, there were 265 GO terms across hundreds of alignments (see below) for which we could both (a) compute meaningful *p*-values according to Sect. 2.4 and (b) have enough predictions to compute a meaningful validation rate for the predictions.

**Table 3:** Correlation between the precision of predictions of *g*-annotations to human proteins in an alignment A versus *g*’s *p*-value in A according to Sect. 2.4

Thresh	N	Pearson	Pearson *p*	$\sigma$’s	Spearman	Spearman *p*	$\sigma$’s
$10^{-2}$	56,470	$-$ 0.371	$9.7\times 10^{-1939}$	94.8	$-$ 0.579	$1.6\times 10^{-6126}$	168.6
$10^{-4}$	49,270	$-$ 0.378	$1.5\times 10^{-1774}$	90.7	$-$ 0.618	$2.8\times 10^{-6551}$	174.3
$10^{-8}$	36,570	$-$ 0.431	$2.1\times 10^{-1793}$	92.2	$-$ 0.681	$8.1\times 10^{-6802}$	177.6
$10^{-16}$	25,871	$-$ 0.449	$8.1\times 10^{-1411}$	80.9	$-$ 0.688	$5.9\times 10^{-5013}$	152.5
$10^{-32}$	16,272	$-$ 0.473	$9.9\times 10^{-1011}$	68.5	$-$ 0.721	$2.8\times 10^{-3801}$	132.8
$10^{-64}$	7688	$-$ 0.621	$1.1\times 10^{-1037}$	69.4	$-$ 0.827	$2.6\times 10^{-3580}$	128.9
$10^{-128}$	4500	$-$ 0.711	$4.1\times 10^{-990}$	67.8	$-$ 0.737	$3.1\times 10^{-1154}$	73.2

The “thresh” column specifies the upper bound on the *p*-value of *g* in a particular alignment A before A’s *g*-annotation predictions are included in that row; N is the number of (A, *g*) pairs that result, across all alignments and GO terms with human proteins as targets. The “Pearson” column is the correlation between (a) the fraction of *g*-annotation predictions that are validated in alignment A and (b) the *p*-value of *g* in the alignment A that produced the predictions; the “Pearson *p*” column is the *p*-value of the Pearson correlation of the previous column; and the $\sigma$’s column is the number of standard deviations represented by the Pearson *p*. The last three columns duplicate the previous three, but for the Spearman correlation. The correlations are negative because the prediction precision increases as *p*-value decreases, as expected (note: the Pearson and Spearman *p*’s technically decrease in significance as *N* decreases, though they remain highly significant throughout.)

**Fig. 3:**
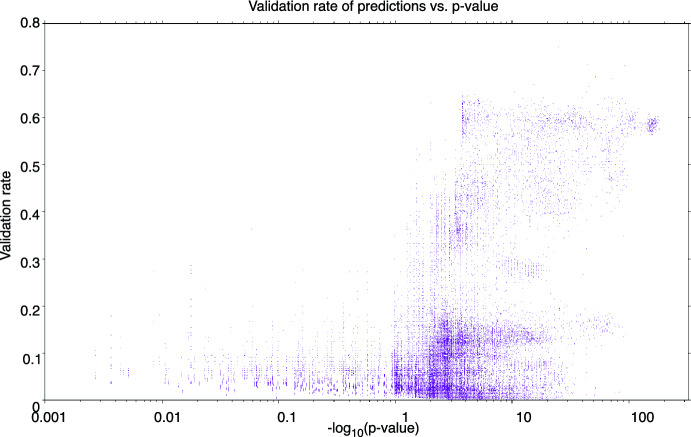
Same data as Table 3, presented as a scatter plot of validation rate of predictions *vs.* *p*-value. Note that *p*-values range from near 1.0 to $10^{-200}$; to clearly represent such an enormous range of *p*-values, we plot the negative of the base-10 logarithm *on a log scale*—that is, the horizontal axis effectively shows the *p*-value having taken the logarithm *twice*: the 0.001 at the far left represents $p =10^{-0.001}\approx 0.9977$ (not significant), while the points at the far right have p-values approaching $10^{-200}$ (highly significant)

To determine the relationship between the *p*-value of Sect. 2.4 and the validation rate of predictions, we conglomerated all predictions across all 265 GO terms across each pairwise network alignment A in which A had a *p*-value with respect to GO term *g* thresholded by the first column of Table 3. As we can see, the correlation is excellent, and gets better as we demand more stringent alignments (smaller value in the “thresh” column). Furthermore, the Spearman correlations are significantly stronger than the Pearsons, because we would not expect the correlation to be *linear* as demanded by Pearson, but only *monotonic* as demanded by Spearman. The same results are presented visually in Fig. 3.

We performed similar tests using the most popular measures from Table 1. In particular, we tested Jaccard similarity, Common GO terms, Normalized Entropy, and Resnik. All have mean prediction precision roughly consistent with the frequency ($\lambda /n$) of the GO term being predicted—ie., the prediction precision is consistent with being random. This result is independent of any threshold placed on the score of the protein pair being used for prediction, or the mean score of the entire alignment.

To conclude: there is a strong correlation between the *p*-value of a network alignment *A* with respect to a particular GO term *g* as computed in this paper, and the validation rate of predicted *g*-annotations from alignment *A*. In other words, as the quality of the alignment increases (smaller *p*-value), its ability to predict new annotations increases. No other currently available GO-based measure of network alignments has this property.

## Discussion

We have presented a method to rigorously compute the *p*-value of matches of a particular GO term in a network alignment. We have rigorously tested the correctness of the method, and demonstrated its biological relevance by showing that higher quality alignments by our measure are better able to *predict* new annotations. No other available GO-based measure has this property.

While our measure is clearly useful, it is not the end of the story. For example, if two very different network alignments both have the same *p*-value for a particular GO term *g*, our method can say nothing about which is “better” with respect to *g*; it would then be the user’s task to look more closely to determine which alignment they prefer.

Given our rigorous *p*-value for each GO term *g* that appears in both networks, one may then wish to compute a GO-based *p*-value of the entire alignment. This requires a method of combining the multitude of “per-GO-term” *p*-values into a single, “holistic” GO-based *p*-value for the entire alignment. Some of the problems are discussed above (cf. Sect. 1.1). While many existing methods suffer the same problem (not accounting for inter-relationships), some existing methods do: Resnik’s semantic similarity score (Resnik 1999) and Mean Normalized Entropy (Liao et al. 2009) use information theoretic/statistical physics arguments to formally account for the inter-relationships between GO terms in the hierarchy—though our tests show that these measures do not correlate with the validation rate of predictions. One could also imagine a combinatorial analysis similar to the one herein, but applied to the GO hierarchy itself rather than to annotations. Doing so rigorously is a challenging problem in itself, and is well beyond the scope of this paper; to our knowledge nobody has yet worked out how to rigorously account for the issues raised in our bulleted list in Sect. 1; see for example surveys (Mistry and Pavlidis 2008; Guzzi et al. 2012; Harispe et al. 2015).

Ultimately, all of the complications of the hierarchy—including even cyclic dependencies—boil down to the simple fact that the appearance of annotations from a GO term $g_1$ may be *correlated* with the appearance of annotations of another GO term $g_2$—or in fact with many such other GO terms. There are known ways to modify *p*-values computed using values with known correlations, but in our case, the exact correlations are unknown and difficult to compute. However, they can be *estimated* from the data, and the recent *Empirical Brown’s Method* (Poole et al. 2016), which we abbreviate as *EBM*, is designed precisely for the case of combining *p*-values between variables whose correlations can only be estimated from the data. Our code (available on GitHub as described elsewhere in this paper) provides the option of using EBM for this purpose.

Our analysis is easily adapted to evaluate network alignments based on any subset of GO terms. For example, one may wish to separately evaluate the three GO hierarchies of *Biological Process* (BP), *molecular Function* (MF), and *Cellular Component* (CC). Additionally, if sequence information plays any role in constructing the network alignment, one should avoid the use of sequence-based GO terms when evaluating that alignment.

## Data Availability

The code described herein, named *REFANGO* is available on GitHub in the author’s NetGO as refango.sh. SANA is also on GitHub, while the BioGRID networks mentioned in the paper are BIOGRID-3.2.101. The output of Refango applied to the 100 alignments per 28 pairs of BioGRID species, along with the resulting predictions and validation rates, can be found at http://sana.ics.uci.edu/Refango-Predictions.7z.

## Footnotes

1 We note that the Jaccard similarity will approximately account for this because *g* will appear in the denominator of some pairs but not appear in any numerator; however Jaccard has other problems, as explained later.

2 The assumption of independence is not entirely unfounded; for example we could choose $g_0$ to be the *Cellular Component* (CC) GO term GO:0005634, which describes the location “nucleus”, and choose the remainder of GO terms to be *molecular functions* (MF) that tend to occur only outside the nucleus. In fact, in the Sept. 2018 release of the GO term database there are over 700 MF GO terms with the following properties: (a) they annotate exactly one protein (ie., each of over 700 GO terms *g* has $\lambda ^g=1$), and (b) for each such GO term, the one protein it annotates is *not* annotated with GO:0005634. The fact that over 700 such GO terms exist make our independence assumption plausible—at least in this artificial scenario.

## References

[CR1] Aladağ AE, Erten C (2013). SPINAL: scalable protein interaction network alignment. Bioinformatics.

[CR2] Alkan F, Erten C (2014). BEAMS: backbone extraction and merge strategy for the global many-to-many alignment of multiple PPI networks. Bioinformatics.

[CR3] Alkan F, Erten C (2015). SiPAN: simultaneous prediction and alignment of protein-protein interaction networks. Bioinformatics.

[CR4] Altschul SF, Gish W, Miller W, Lipman DJ (1990). Basic local alignment search tool. J Mol Biol.

[CR5] Balomenos AD, Tsakanikas P, Manolakos ES (2015) Tracking single-cells in overcrowded bacterial colonies. In: 2015 37th annual international conference of the IEEE engineering in medicine and biology society (EMBC), pp 6473–6476. 10.1109/EMBC.2015.7319875 10.1109/EMBC.2015.7319875 26737775

[CR6] Chindelevitch L, Ma CY, Liao CS, Berger B (2013). Optimizing a global alignment of protein interaction networks. Bioinformatics.

[CR7] Clark C, Kalita J (2014). A comparison of algorithms for the pairwise alignment of biological networks. Bioinformatics.

[CR8] Clark C, Kalita J (2015). A multiobjective memetic algorithm for PPI network alignment. Bioinformatics.

[CR9] Crawford J, Sun Y, Milenković T (2015). Fair evaluation of global network aligners. Algorithms Mol Biol.

[CR10] Djeddi WE, Yahia SB, Nguifo EM (2018). A novel computational approach for global alignment for multiple biological networks. IEEE/ACM Trans Comput Biol Bioinform.

[CR11] Elmsallati A, Msalati A, Kalita J (2018). Index-based network aligner of protein-protein interaction networks. IEEE/ACM Trans Comput Biol Bioinform TCBB.

[CR12] Faisal FE, Meng L, Crawford J, Milenković T (2015). The post-genomic era of biological network alignment. EURASIP J Bioinf Syst Biol.

[CR13] Fan J, Cannistra A, Fried I, Lim T, Schaffner T, Crovella M, Hescott B, Leiserson MD (2019). Functional protein representations from biological networks enable diverse cross-species inference. Nucleic Acids Res.

[CR14] Flannick J, Novak A, Srinivasan BS, McAdams HH, Batzoglou S (2006). Graemlin: general and robust alignment of multiple large interaction networks. Genome Res.

[CR15] Gligorijević V, Malod-Dognin N, Pržulj N (2015) FUSE: multiple network alignment via data fusion. Bioinformatics btv731 10.1093/bioinformatics/btv731 26668003

[CR16] Gong M, Peng Z, Ma L, Huang J (2015). Global biological network alignment by using efficient memetic algorithm. IEEE/ACM Trans Comput Biol Bioinf.

[CR17] Guzzi PH, Milenković T (2017) Survey of local and global biological network alignment: the need to reconcile the two sides of the same coin. Brief Bioinform bbw132 10.1093/bib/bbw132 28062413

[CR18] Guzzi PH, Mina M, Guerra C, Cannataro M (2012). Semantic similarity analysis of protein data: assessment with biological features and issues. Brief Bioinform.

[CR19] Harispe S, Ranwez S, Janaqi S, Montmain J (2015). Semantic similarity from natural language and ontology analysis. Synth Lect Hum Lang Technol.

[CR20] Hashemifar S, Xu J (2014). HubAlign: an accurate and efficient method for global alignment of protein-protein interaction networks. Bioinformatics.

[CR21] Hashemifar S, Ma J, Naveed H, Canzar S, Xu J (2016). ModuleAlign: module-based global alignment of protein-protein interaction networks. Bioinformatics.

[CR22] Hashemifar S, Huang Q, Xu J (2016). Joint alignment of multiple protein-protein interaction networks via convex optimization. J Comput Biol.

[CR23] Hu J, Kehr B, Reinert K (2014). NetCoffee: a fast and accurate global alignment approach to identify functionally conserved proteins in multiple networks. Bioinformatics.

[CR24] Kalecky K, Cho YR (2018). PrimAlign: PageRank-inspired Markovian alignment for large biological networks. Bioinformatics.

[CR25] Kazemi E, Hassani H, Grossglauser M, Modarres HP (2016). PROPER: global protein interaction network alignment through percolation matching. BMC Bioinform.

[CR26] Kuchaiev O, Pržulj N (2011). Integrative network alignment reveals large regions of global network similarity in yeast and human. Bioinformatics.

[CR27] Kuchaiev O, Milenković T, Memišević V, Hayes W, Pržulj N (2010). Topological network alignment uncovers biological function and phylogeny. J R Soc Interface.

[CR28] Liao CS, Lu K, Baym M, Singh R, Berger B (2009). IsoRankN: spectral methods for global alignment of multiple protein networks. Bioinformatics.

[CR29] Malod-Dognin N, Pržulj N (2015). L-GRAAL: Lagrangian graphlet-based network aligner. Bioinformatics.

[CR30] Malod-Dognin N, Ban K, Pržulj N (2017). Unified alignment of protein-protein interaction networks. Sci Rep.

[CR31] Mamano N, Hayes WB (2017). SANA: simulated annealing far outperforms many other search algorithms for biological network alignment. Bioinformatics.

[CR32] Milano M, Guzzi PH, Cannataro M (2018). Glalign: A novel algorithm for local network alignment. IEEE/ACM Trans Comput Biol Bioinf.

[CR33] Milenković T, Ng WL, Hayes W, Pržulj N (2010). Optimal network alignment with graphlet degree vectors. Cancer Inform.

[CR34] Mir A, Naghibzadeh M, Saadati N (2017). INDEX: incremental depth extension approach for protein-protein interaction networks alignment. Biosystems.

[CR35] Mistry M, Pavlidis P (2008). Gene Ontology term overlap as a measure of gene functional similarity. BMC Bioinform.

[CR36] Neyshabur B, Khadem A, Hashemifar S, Arab SS (2013). NETAL: a new graph-based method for global alignment of protein-protein interaction networks. Bioinformatics.

[CR37] Patro R, Kingsford C (2012). Global network alignment using multiscale spectral signatures. Bioinformatics.

[CR38] Pesquita C, Faria D, Bastos H, Ferreira AE, Falcão AO, Couto FM (2008). Metrics for GO based protein semantic similarity: a systematic evaluation. BMC Bioinform.

[CR39] Pesquita C, Faria D, Falcao AO, Lord P, Couto FM (2009). Semantic similarity in biomedical ontologies. PLoS Comput Biol.

[CR40] Poole W, Gibbs DL, Shmulevich I, Bernard B, Knijnenburg TA (2016). Combining dependent P-values with an empirical adaptation of Brown’s method. Bioinformatics.

[CR41] Resnik P (1995) Using information content to evaluate semantic similarity in a taxonomy. In: Proceedings of the 14th international joint conference on artificial intelligence—volume 1, IJCAI’95. Morgan Kaufmann Publishers Inc., San Francisco, pp 448–453. http://dl.acm.org/citation.cfm?id=1625855.1625914

[CR42] Resnik P (1999). Semantic similarity in a taxonomy: an information-based measure and its application to problems of ambiguity in natural language. J Artif Intell Res JAIR.

[CR43] Sarajlić A, Malod-Dognin N, Yaveroğlu ÖN, Pržulj N (2016). Graphlet-based characterization of directed networks. Sci Rep.

[CR44] Saraph V, Milenković T (2014). MAGNA: maximizing accuracy in global network alignment. Bioinformatics.

[CR45] Schlicker A, Domingues FS, Rahnenführer J, Lengauer T (2006). A new measure for functional similarity of gene products based on Gene Ontology. BMC Bioinform.

[CR46] Singh R, Xu J, Berger B (2008). Global alignment of multiple protein interaction networks with application to functional orthology detection. Proc Natl Acad Sci.

[CR47] Sun Y, Crawford J, Tang J, Milenkovic T (2015) Simultaneous optimization of both node and edge conservation in network alignment via WAVE. In: Pop M, Touzet H (eds) Algorithms in bioinformatics. Lecture notes in computer science, vol 9289. Springer, Berlin, pp 16–39. 10.1007/978-3-662-48221-6_2

[CR48] The Gene Ontology Consortium (2008) Nucleic Acids Res 36(suppl 1):D440 10.1093/nar/gkm883 PMC2238979 17984083

[CR49] Vijayan V, Milenković T (2018). Multiple network alignment via multiMAGNA++. IEEE/ACM Trans Comput Biol Bioinform.

[CR50] Wang S, Atkinson GR, Hayes WB (2022). SANA: cross-species prediction of Gene Ontology GO annotations via topological network alignment. Nat Partner J Syst Biol Appl.

[CR51] Wang S, Chen X, Frederisy BJ, Mbakogu BA, Kanne AD, Khosravi P, Hayes WB (2022) On the current failure—but bright future—of topology-driven biological network alignment. Protein Interact Netw 21(1) 10.1016/bs.apcsb.2022.05.005 35871888

[CR52] Xie J, Xiang C, Ma J, Tan J, Wen T, Lei J, Nie Q (2016). An adaptive hybrid algorithm for global network alignment. IEEE/ACM Trans Comput Biol Bioinform TCBB.

[CR53] Zhu Y, Li Y, Liu J, Qin L, Yu JX (2017) GMAlign: a new network aligner for revealing large conserved functional components. In: 2017 IEEE international conference on bioinformatics and biomedicine (BIBM) (IEEE), pp 120–127

